# Personality Comparison between Lethal and Non-lethal Intimate Partner Violence Perpetrators and Their Victims

**DOI:** 10.1007/s11121-023-01619-w

**Published:** 2023-11-25

**Authors:** David Pineda, Manuel Galán, Ana Martínez-Martínez, Pablo J. Andrés-Prades, Nestor García-Barceló, Enrique J. Carbonell, José L. González-Álvarez

**Affiliations:** 1 Forensic Psychology Unit, Miguel Hernández University of Elche, Avda. de La Universidad, 03202 S/N. Edf. AltamiraElche, Alicante Spain; 2 Psychology Department, Faculty of Medicine, Catholic University of Murcia, Av. de los Jerónimos, 135, 30107 Guadalupe de Maciascoque, Murcia, Spain; 3 C/ Francisco Tomás y Valiente, Escuela Politécnica Superior, Institute for Forensic and Security Sciences (ICFS) of the Autonomous University of Madrid, Ciudad Universitaria Cantoblanco, Edificio C, Despacho C-302, 28049 Madrid, Spain; 4 Central Departmental Building/Office 1P03, University Research Institute of Criminology and Criminal Science, School of Law, University of Valencia, Tarongers Campus, 46071 Valencia, Spain; 5 Dirección General de Coordinación y Estudios, Secretaría de Estado de Seguridad, Madrid, Spain

**Keywords:** Personality, Neuroticism, Extraversion, Psychoticism, Intimate partner violence against women, Intimate partner femicide

## Abstract

Intimate partner violence against women (IPVAW) and femicide (intimate partner femicide, IPF), as a worldwide phenomenon, cannot be explained in a simple way. From an ecological point of view, there are individual factors contemplated. In the current studies, we consider personality as an individual factor to clarify what differentiates a non-lethal IPVAW situation from a femicide. Study 1 was designed to investigate the accuracy with which trained interviewers judged the personality of a group of IPVAW perpetrators during an interview. The target sample of study 1 was composed of 293 males who after being interviewed completed a measure of personality assessing the “Big Three” model of personality. The interviewers performed fairly accurate judgements about the personality of the target participants. Study 2 shows the differences in personality, using Eysenck’s personality model, between the IPF and IPVAW perpetrators and their victims. The total sample study 2 was formed of 551 participants distributed among IPF perpetrators, IPVAW perpetrators, and the victims of both groups. Differences in proportions were observed between both groups of perpetrators as well as between each group and their respective victims. With these findings, we propose personality as a femicide risk factor that should be taken into consideration by police officers and other practitioners when receiving an IPVAW report.

## Introduction

Intimate partner violence against women (IPVAW) is a common type of violence, even considered recently as a public health problem (Organización Mundial de la Salud [OMS], 2013). Inside this frame, violence against women is described by the United Nations (1993, p.2) as “any act of gender-based violence that results in, or is likely to result in, physical, sexual, or mental harm or suffering to women, including threats of such acts, coercion or arbitrary deprivation of liberty, whether occurring in public or in private life”. Prior research on batterer typologies has provided insights into the heterogeneous nature of IPVAW and, thus, into the possibility of providing more accurate population-based interventions, risk management procedures, and police and judicial measures, based on the characteristics of the offenders (González-Álvarez et al., 2021).

IPVAW, even though considered a different phenomenon from intimate partner femicide (IPF), can eventually finish with the death of the female partner (Pineda, Galán et al., 2023). In the Spanish context, it is alarming to note that approximately 15% of women have experienced some form of physical or sexual violence, with approximately 30% reporting incidents of psychological violence. However, a stark reality emerges when examining homicide statistics. Less than 20% of overall homicide victims are women, but within this percentage, over half of them fell victim to their partner, former partner, or another relative (United Nations Office on Drugs and Crime, 2019). This underscores the deeply concerning prevalence of intimate partner violence. Kivisto (2015) sheds further light on this issue, revealing that women are six times more likely to be victims of homicide than men. Specifically, within the Spanish context, 51% of all femicides in Spain were perpetrated by their partner or former partner.

Following the ecological perspective developed by various authors, IPVAW would appear as a result of aggregated risk factors organized in multiple levels (Heise, 1998; Krug et al., 2002). A risk factor is understood as any aspect that correlates with a possible result without the necessity of a direct cause-effect relationship, just increasing the likelihood of occurrence (López-Ossorio et al., 2017). Opposite to risk factors, protective factors take place. These are the ones that make the likelihood of occurrence decrease, for example, the distance between victim and perpetrator (Bonta & Andrews, 2016). Inside this classification, furthermore, we can distinguish between static and dynamic factors, where statics are those developed from the own history of the person and thus unchangeable and dynamics are those that are susceptible to change over time such as a personal situation or couple’s affective state (Bonta & Andrews, 2016).

To unravel the complexity of IPVAW, López-Ossorio et al. (2017) delineated four distinct risk levels. These encompass individual factors, including personal experiences during childhood, health issues, substance abuse, and exposure to violence. Moving to the family level, it includes aspects such as traditional upbringing, diminished marital satisfaction, and lower educational attainment. Expanding further, the community level introduces elements such as the normalization of violence and the absence of institutions addressing IPV. Finally, at the societal level, gender-related factors come into play, encompassing traditional associations between masculinity and violence, as well as the unequal societal position of women (López-Ossorio et al., 2018). Considering IPF, certain specific risk indicators have also been located differently from those identified for IPVAW (Cunha & Goncalves, 2016; Pineda, Galán et al., 2023). Some of the variables that differ are related to economic or work-related problems, psychopathology, or the existence of other different stressors in the perpetrator as being in the process of separation from the victim (López-Ossorio et al., 2021; Pineda, Galán et al., 2023).

Delving specifically into individual factors, one of the most contemplated aspects at this level is the personality, understood as the individual set of characteristics observable in different patterns of feelings, behaviours, and thoughts (Cervone & Lawrence, 2018; Goldberg, 1993). One of the original and thus largely studied models of personality is the PEN model (Eysenck & Eysenck, 1975). This model, understood from a dimensional perspective, is based on three main traits: psychoticism as opposed to “normality”, extraversion in the opposite pole to introversion, and neuroticism in relation to emotional stability (Eysenck & Eysenck, 1985). Psychoticism, as the most antisocial of the three traits, is characterized by a lack of empathy, aggressiveness, and contravening stipulated social norms. Extraversion describes a pattern of personality with a tendency to interact with other people and exteriorise their feelings. The last one, neuroticism, reflects a personality with high levels of stress, affectivity, and a negative approach to life (Eysenck & Eysenck, 1968, 1975).

There is previous literature linking IPVAW with different models of personality. For example, Pineda et al. (2021) found that those people who perpetrated more virtual abusive behaviours towards their partners tend to present higher scores in traits like subclinical psychopathy or everyday sadism. Another example is the research conducted by Ulloa et al. (2016) who mentioned that traits like openness, extraversion, and neuroticism were connected with these types of behaviors. The investigation has extended its focus beyond the perpetrators to include an examination of the victims in the context of IPVAW from a dyadic approach (e.g. Juarros-Basterretxea et al., 2022). Within the context of personality, prior research has shown that some traits (such as neuroticism or sadism) can function as risk and protective factors for both actors, victims, and perpetrators (Juarros-Basterretxea et al., 2022; Pineda, Martínez-Martínez et al., 2023).

However, there is just one pilot investigation using the PEN model of personality. This pilot investigation conducted using just one part of the sample suggests that IPF perpetrators presented high scores in psychoticism, low in extraversion, and high in neuroticism, while their victims tended to present low scores in psychoticism, high in extraversion, and low in neuroticism (García-Barceló et al., 2018). Notwithstanding, there is extensive literature that links the PEN model of personality and criminality (e.g. Dunlop et al., 2012; Eysenck et al., 1977). This previous research reflects that the psychoticism trait is the most related personality factor of this model to different antisocial and criminal outcomes. However, other investigations have shown that also high levels of extraversion and neuroticism can be related to different criminal typologies (Levine & Jackson, 2004; Naqvi & Kamal, 2013; van Dam et al., 2007). Furthermore, research within other personality models also highlights the significance of the neuroticism or emotional stability factor in relation to these outcomes (Dunlop et al., 2012; Sánchez-Teruel & Robles-Bello, 2013).

Since, from this ecological perspective, personality is considered an IPVAW risk factor, its assessment should be considered (Ulloa et al., 2016). There are several ways to assess personality, which could be broadly summarized in direct and indirect personality assessment methods (Mischel, 1972). Direct personality assessment tends to be considered the gold standard in the evaluation of personality since it implies the use of standardized, validated questionnaires that offer an objective and evaluator-independent score. In this style of evaluation, the assessed people know that they are been evaluated (Kyllonen & Kell, 2018). An example of these questionnaires is the Eysenck Personality Questionnaire (EPQ; Eysenck & Eysenck, 1975), which measures the previously described traits of the PEN model. Furthermore, as mentioned before, personality can also be measured indirectly by not using standardized tools but by observing, obtaining information, and analyzing cues about the different target traits of the assessed person (Ault, 2017).

Indirect personality assessment as the ability to accurately identify other people’s personality profiles is part of a broader construct named interpersonal accuracy (Hall et al., 2016). It is called indirect because it does not necessarily need the collaboration of the target person to perform it (González-Álvarez et al., 2015; Sotoca et al., 2019). This assessment strategy employs various techniques, such as conducting open interviews with the subject or their acquaintances and observing their actions and behaviours. These methods are used to comprehensively analyze all available information about the subject and develop a profile or ascertain some of their key traits (Ault, 2017; Muñoz-Espinosa & Santos-Hermoso, 2020).

Indirect personality assessment methods are well established and serve as invaluable, if not essential, tools in several contexts (Allik et al., 2016). For instance, they prove highly beneficial in criminal investigations, aiding in interviews, suspect interrogations to gather additional information, during negotiations, or even in predicting potential behaviours of individuals (Ault, 2017; Muñoz-Espinosa & Santos-Hermoso, 2020). One main context in which indirect assessment is necessary is when the assessed person has deceased (i.e. the psychological autopsy) (Aquila et al., 2018). A psychological autopsy is a methodological approach used in forensic psychology that seeks to reconstruct or obtain information about different psychological variables of an individual who has died (e.g. emotional states, personality traits). This investigative technique involves a comprehensive examination of various sources of information, including medical records, personal documents, and interviews with family and friends, among others (Isometsä, 2001).

However, an important drawback of personality assessment using indirect tools is that it might vary from one perceiver to another. Theoretical models such as the lens model and a variant of it, the realistic accuracy model, have been developed to explain why perceivers are or are not accurate (Funder, 2012; Karelaia & Hogarth, 2008). The basis of these models relies on the idea that there are different, observable, and valid personality cues (e.g. an extravert person tends to be more talkative and expressive, or a neurotic might express some degree of frustration or use negative self-references) that the perceiver should detect and use them to make an accurate judge (Back & Nestler, 2016; Eysenck & Eysenck, 1975; Nestler & Back, 2013). Thus, it is important to identify the variables that result in an accurate judgement of someone’s personality to control them (Back & Nestler, 2016).

In this line, personality research has identified some moderators that influence the accuracy of the judgements, which are named: “good trait”, traits that are more observable than others (Connelly & Ones, 2010); “good information”, the more information, the better (Letzring & Human, 2014); “good judge”, where it has been suggested that people with a background in behavioural sciences and better emotion recognition might be better in this task (Ault, 2017; Back & Nestler, 2016); “good target” which refers to those people who are more expressive and generate more valid cues (Biesanz, 2010; Human et al., 2014); and its interactions (Funder, 2012).

## The Present Study

The present study seeks to explore the differences in personality between lethal IPVAW perpetrators and non-lethal ones to deeply understand this risk factor at an individual level what makes someone commit homicide or aggression. Also, understanding IPVAW from a dyadic approach (Leone et al., 2016; Sommer et al., 2017), we aim to explore the differences between these perpetrator personalities and their victims and between both groups of victims as has been done in previous studies with other different characteristics of these populations (e.g., Pineda, Galán, et al., 2023; Pineda, Rico-Bordera, et al., 2023). Furthermore, due to its usefulness in this applied field, we aim to add research to the interpersonal accuracy field, by testing the accuracy of trained judges in assessing personality indirectly in non-laboratory conditions.

With the purpose of addressing the stated objectives, and based on the previous literature, we formulated the following hypothesis (*H*):

*H1.* The interviewers will perform accurate judgements of the target’s personality.

*H2.* Attending to personality, lethal perpetrators will present higher scores in psychoticism and neuroticism, while non-lethal perpetrators will display higher scores in extraversion, compared to each other. Also, they will show differences in these traits with their victims.

### Study 1

Study 1 was specifically designed to evaluate whether the trained interviewers were or were not accurate in assessing victims’ and perpetrators’ personalities. It was performed to provide a methodological basis for the personality assessment procedure in study 2.

### Method

The present study is part of a research project carried out by the national team for the detailed review of intimate partner homicides against women coordinated by the Secretary of State for Security of the Ministry of the Interior (see González et al., 2018) and carried out between 2015 and 2021. To carry out this project, the mentioned team counted on the collaboration of three other government agencies at the national level in Spain, the General Prosecutor’s Office for Violence against Women, the General Council of the Judiciary and the Government Delegation against Gender Violence, and the State Security Forces and Corps, and the penitentiary institutions of 28 provinces in Spain, 21 Spanish universities, and 3 scientific institutions (González-Álvarez et al., 2023).

#### Participants

##### Judges

The judges of this study were master’s degree students in forensic psychology and forensic criminology.

##### Targets

Two hundred ninety-three convicted for gender-based violence served as the targets for this study. The age of the participants ranged between 18 and 85 years (*M* = 42.01, *SD *= 13.90). Of them, 75.4% were Spanish, and all of them were male. Participants did not receive any benefits for participating in the study.

The target participants were recruited using stratified sampling from Spanish police records, consulting the VioGen system (González Álvarez et al., 2018), and after selecting the specific participants randomly. The selection criteria were involved in an IPVAW sentence either lethal or non-lethal. The participants included in this study were the non-lethal perpetrators.

#### Measures

The indirect assessment of personality was completed by filling out an ad hoc* personality checklist* based on the PEN model of personality (Eysenck & Eysenck, 1968, 1975). The judges had to choose in the three supertraits whether the participant displayed a high or a low level of each of them. Also, the option “not known” was available, to avoid forcing a random choice.

As a direct tool for assessing personality, the *abbreviated form of the Revised Eysenck Personality Questionnaire* (EPQR-A; Francis et al., 1992) was used, which is based on the original EPQ (Eysenck & Eysenck, 1975). The version applied in this study was the Spanish adaptation of the EPQR-A (Sandín et al., 2002). It is composed of 24 items and 4 subscales, with a yes/no type of answer. The reliability coefficients (*α*) in the original Spanish sample for neuroticism and extraversion were 0.78 and 0.74, respectively. Not so good alpha coefficients for the other two scales are as follows: 0.63 for psychoticism and 0.54 for honesty (Sandín et al., 2002).

#### Procedure

The judges who were previously trained to detect valid cues related to the PEN model of personality (for more information, see Muñoz-Espinosa & Santos Hermoso, 2020) conducted a semi-structured interview based on the “manual of action for the review of gender-based homicides of the national team for the detailed review of gender-based homicides” (González et al., 2018) with the target perpetrator. All the interviews were performed by two judges, one leading the interview and the other one listening. The judges had to retrieve exhaustive information about the perpetrator’s lifestyle before the crime and about the day of the conflict for which the first complaint was made. During the interview, of approximately 2 h, the judges had to decide if the targets showed high or low levels of the PEN traits. Each target participant was assessed by two psychologists (the judges) who independently made an indirect assessment of their personality traits and decided together which option to select in the checklist by consensus (Asua, 2006). When the interview was finished, the target participants completed the EPQR-A. The answers offered by the target participant to the EPQR-A were not corrected by the judges with the objective of not biasing their indirect personality judgements.

#### Data Analyses

To study the agreement between the judgements’ assessment of personality and the EPQR-A scores, bivariate correlations were used. Descriptive statistics and bivariate correlations were obtained using SPSS version 23. To ensure the accuracy of the correlations of interest, corrections were applied to account for measurement error attenuation (Carroll et al., 2006). Following Cohen’s suggestion, we assume that small effect size *r* coefficients are larger than 0.10, medium between 0.30 and 0.50, and large over 0.50 (Cohen, 1988).

#### Results

Table 1 shows the correlations between the interviewer’s personality judgement and the scores obtained by the target participants in the EPQR-A. Large and medium correlations were found for the extraversion and neuroticism traits, respectively, while a small correlation was found for the psychoticism variable. Small but significant correlations were also observed between the sincere scale and the psychoticism ones.

**Table 1:** Bivariate correlations between the types of personality assessment

	(1)	(2)	(3)	(4)	(5)	(6)	(7)
(1) Neuroticism (judges)	1						
(2) Extraversion (judges)	− 0.13*	1					
(3) Psychoticism (judges)	0.25**	− 0.11	1				
(4) Neuroticism (EPQR-A)	0.41**	− 0.02	0.13	1			
(5) Extraversion (EPQR-A)	− 0.09	0.55**	− 0.11	− 0.19*	1		
(6) Psychoticism (EPQR-A)	0.08	− 0.01	0.23**	0.13	− 0.18*	1	
(7) Sincerity (EPQR-A)	0.07	− 0.15	0.18*	0.18*	0.30*	0.25*	1

*p < 0.05, **p < 0.01

Correcting the observed relationships for measurement error due to attenuation, we obtain higher correlations between the interviewers’ judgements and the EPQR-A results for the three traits: neuroticism (*r* = 0.46, *p* < 0*.*01), extraversion (*r* = 0.23, *p* < 0.05), and psychoticism (*r* = 0.23, *p* < 0.01).

#### Study 1 Discussion

The main objective of this study was to offer a methodological baseline for study two by testing the accuracy of the interviewers in assessing personality. The results obtained partially confirmed the *H1* showing that interviewers made accurate judgements about the personality of the target participants, offering a substantial baseline for study 2. The “good trait” moderator is clearly observable in our results (Funder, 2012). In this study, our interviewers were more accurate in retrieving cues of the more easily observable traits, thus making more accurate predictions in extraversion and not so accurate in psychoticism (Connelly & Ones, 2010). Furthermore, the difficulties or the differences in the assessment of psychoticism, against the other traits, might be explained by the sincerity levels of the participants: those who are more sincere might be more prone to reveal or show behaviours that can be considered less socially desirable, making this trait more difficult to recognize in insincere individuals (Galán et al., 2023).

At this point, it is also important to remark that these differences between the direct and the indirect assessment of personality could be also explained by the self-other knowledge asymmetry (SOKA) model. This model proposed by Vazire et al.’s (2010) claims that there are some aspects of one’s personality which are better known by other people; meanwhile, others should be recognized in a superior way by oneself, leading to judgement discrepancies (Neubauer et al., 2018). For example, literature about the personality traits developed based on the SOKA model has shown that there is a tendency to obtain higher levels of agreement in the more visible traits such as extraversion (Lee & Ashton, 2017).

Notwithstanding, the study shows that generally, the interviewers were able to retrieve more valid cues—“good information”—the easier the traits were (i.e. extraversion), while the judgements made over those traits with more difficult cues to retrieve (i.e. psychoticism) have to be considered with caution (Letzring & Human, 2014). However, it is also worth mentioning that the psychoticism trait collects a wide variety of behaviours and tendencies making it harder to conceptualize and measure in self-report scales (attending to its internal consistency values) (Cale, 2006; Sandín et al., 2002).

Furthermore, albeit the research about who is a “good judge” is not very consistent, our results suggest that at least those who have a bachelor’s graduate degree (in psychology or criminology) and are trained for retrieving cues emit judgements that tend to correlate with the results found in self-report measures—the gold standard in personality assessment (Ault, 2017; Back & Nestler, 2016).

### Study 2

The main purpose of study 2 is to investigate the differences or similarities in personality between IPF perpetrators and non-lethal IPVAW perpetrators and to explore these same variables in the victims. In like manner, we pursue to observe the differences in personality between the perpetrators and their victims.

#### Method

This second study is also part of the research project carried out by the national team for the detailed review of intimate partner homicides against women coordinated by the Secretary of State for Security of the Ministry of the Interior mentioned and described in study 1.

#### Participants

Participants were recruited following the same procedure as the target participants in study 1. The total sample of study 2 was formed by *N* = 551 participants divided into four groups. On the one hand, from the IPF group, we obtained a subsample of *n* = 169 perpetrators with an average age of 46.17 (*SD* = 14.63) and a subsample of *n* = 165 deathly victims with an average age of 41.87 (*SD* = 14.62). From this IPF subsample, 71.9% of the perpetrators and 68.4% of the victims were Spanish. The selection of these participants was made following the quota sampling method based on the percentages of convicted femicides in Spain (Gónzalez-Álvarez et al., 2019). On the other hand, the group of non-lethal perpetrators was composed of *n* = 110 and some of their victims *n* = 107. This sample of non-lethal perpetrators was composed of 80.9% Spanish participants with an average age of 35.96 (*SD* = 10.82). And their victims were 83.6% from Spain, with an average age of 32.97 (*SD* = 10.34).

#### Measures

As in study 1, the indirect assessment of personality was completed by filling an ad hoc* personality checklist* based on the PEN model of personality. The interviewers had to choose between “high”, “low”, or “not known” to fulfil the answer.

#### Procedure

The procedure followed in study 2 was similar to study 1, where all the alive participants answered the same semi-structured interview. In those cases, concerning lethal victims and perpetrators who committed suicide (25%), the method to assess their personalities was the psychological autopsy (defined in the introduction). In this case, besides the collection of all the available information from the police records, interviews were conducted with at least five relatives or close friends of the deceased. These interviews aimed to gather accurate insights into the victim’s personality, mirroring the information collected from living participants. This comprehensive approach ensures a thorough understanding of the victim’s personality within the context of the psychological autopsy.

#### Data Analyses

Data analyses for descriptive statistics and group differences were conducted with SPSS, version 23. Group differences were compared using chi-square tests. The significance level was corrected using the Bonferroni correction to account for an amplified alpha, which is the result of dividing the *α* (0.05) by the number of analyses performed (20); hence, a significant effect was considered if *p* < 0.0025.

#### Results

First, Table 2 compares the personality, between the IPF perpetrators group and the non-lethal IPVAW perpetrators group. Significant differences (*p* < 0.0025, Bonferroni fit) in the proportions are displayed in all the variables. The highest difference between IPF perpetrators and IPVAW perpetrators was found in the psychoticism trait with a higher proportion of IPF perpetrators (75.6%) presenting high scores on this trait compared to the IPVAW group (42.2%).

**Table 2:** Frequencies and chi-square results for personality and substance consumption in the perpetrators

		IPF perpetrator		IPVAW perpetrator		*χ*^*2*^ (1)
		*n*	%	*n*	%	
Neuroticism	Low	41	25.5	52	47.3	13.79*
	High	120	74.5	58	52.7	
Extraversion	Low	94	58	36	33	16.31*
	High	68	42	73	67	
Psychoticism	Low	39	24.4	63	57.8	30.77*
	High	121	75.6	46	42.2	

^*^p < 0.0025 (Bonferroni fit)

Table 3 shows the comparison between the IPF victims group and the non-lethal IPVAW victims group. There were no significant differences in any of the traits compared.

**Table 3:** Frequencies and chi-square results for personality and substance consumption in the victims

		IPF victim		IPVAW victim		*χ*^*2*^ (1)
		*n*	%	*n*	%	
Neuroticism	Low	77	49	26	37.7	2.50
	High	80	51	43	62.3	
Extraversion	Low	36	22.8	26	37.7	5.37
	High	122	77.2	43	62.3	
Psychoticism	Low	125	80.1	61	88.4	2.29
	High	31	19.9	8	11.6	

^*^p < 0.0025 (Bonferroni fit)

When comparing the IPF perpetrators with their victims (Table 4), significant differences are observed in all the personality traits (*p* < 0.0025). With bigger differences in the psychoticism (*χ*^*2*^_(1)_ = 98.35, *p* < 0.0025) and extraversion traits (*χ*^*2*^_(1)_ = 41.18, *p* < 0.0025). In the case of the extraversion trait, the proportion of victims rated as high in this trait (77.2%) was bigger than the proportion of perpetrators rated in the same way (42.0%).

**Table 4:** Frequencies and chi-square results for personality and substance consumption in the lethal IPVAW group

		IPF perpetrator		IPF victim		*χ*^*2*^ (1)
		*n*	%	*n*	%	
Neuroticism	Low	41	25.5	77	49	18.94*
	High	120	74.5	80	51	
Extraversion	Low	94	58	36	22.8	41.18*
	High	68	42	122	77.2	
Psychoticism	Low	39	24.4	125	80.1	98.35*
	High	121	75.6	31	19.9	

^*^p < 0.0025 (Bonferroni fit)

The proportions in the psychoticism trait (*χ*^*2*^_(1)_ = 18.73, *p* < 0.0025) were higher for the non-lethal IPVAW perpetrators (42.2%) compared to their victims (11.6%) (Table 5).

**Table 5:** Frequencies and chi-square results for personality and substance consumption in the non-lethal IPVAW group

		IPVAW perpetrator		IPVAW victim		*χ*^*2*^ (1)
		*n*	%	*n*	%	
Neuroticism	Low	52	47.3	26	37.7	1.59
	High	58	52.7	43	62.3	
Extraversion	Low	36	33	26	37.7	0.40
	High	73	67	43	62.3	
Psychoticism	Low	63	57.8	61	88.4	18.73^*^
	High	46	42.2	8	11.6	

^*^p < 0.0025 (Bonferroni fit)

#### Study 2 Discussion

The main objective of study 2 was to investigate the differences in personality between lethal and non-lethal IPVAW perpetrators and their victims, being the last objective to establish differentiated patterns that reflect the personality profiles of the perpetrators and their victims.

Starting with the differences in the personality profiles, we have found several differences in the three traits between the studied groups. First, supporting *H2*, lethal perpetrators present higher levels of neuroticism and psychoticism traits than non-lethal ones. These findings were expected since personalities with high levels of neuroticism tend to present elevated levels of anxiety, be worried, and have difficulties coping with emotions. Furthermore, the psychoticism trait is conceived as the most antisocial of the three traits, related to aggressiveness or lack of empathy (Eysenck & Eysenck, 1968, 1985). Finally, also in accordance with *H2*, regarding the extraversion trait, the non-lethal IPVAW group is the one displaying higher levels. And no differences were found between the victims.

Attending to the differences in personality inside the couples, for the non-lethal IPVAW participants, our results show similar levels in the proportions of the evaluated traits excluding psychoticism where the perpetrators present higher scores. On the contrary, the proportions displayed in the IPF couples showed differences between the three traits. In this case, the perpetrator group showed higher proportions of high scores in psychoticism and neuroticism while smaller in extraversion compared to their victims. Again, as expected, those traits more related to antisocial behaviour and emotional instability appear more frequently in the perpetrators than in the victims (Davoren et al., 2017; Eysenck & Eysenck, 1968; Galán et al., 2023).

## General Discussion

The main objective of the current investigation was to explore the differences in personality among lethal and non-lethal IPVAW perpetrators and victims. Furthermore, we aimed to show how trained interviewers were able to make accurate judgements about others’ personality, because in applied environments, the direct assessment of the personality is not usually possible, especially when there are dead persons involved. In this regard, our results showed that the interviewers provided sufficiently accurate personality assessments.

Attending to the differences in personality between groups, the lethal perpetrators tend to present higher levels in the psychoticism and neuroticism dimensions while lower levels in extraversion, compared to the non-lethal group of perpetrators. The lethal perpetrators tend to show higher levels of anxiety and an odd or bizarre way of acting and thinking (Eysenck & Eysenck, 1985). These findings are convergent with previous literature stating that those disorders related to an anxious way of behaving and thinking and those characterized by eccentric behaviour tend to be more related to this type of lethal perpetrators (Liem & Koenraadt, 2008). Regarding the extraversion personality dimension, as asserted, the IPF group displays lower levels. As explained by López et al. (2016; p.11), “When a high score in neuroticism is combined with a low score in extraversion, the person tends to be very anxious, worried, pessimistic, negative, with low self-esteem and with a tendency to depression”. This definition presents some characteristics associated with personality disorders such as paranoid, avoidant, or obsessive–compulsive, disorders that tend to be related to lethal IPVAW perpetrators (Belfrage & Rying, 2004; Liem & Koenraadt, 2008).

Additionally, by combining these results, we obtain a personality pattern for the lethal IPF perpetrators characterized by high neuroticism, high psychoticism, and low extraversion, related to the “stress-accumulator” personality type (Eysenck & Eysenck, 1985; Gray, 1970). However, this does not imply that non-lethal IPVAW perpetrators cannot display similar patterns of personality too (Loinaz et al., 2018).

Attending to the differences in personality between perpetrators and victims, the perpetrators tend to present higher scores on the psychoticism scale than their victims. This finding, again, is convergent to the nature of psychoticism as the most antisocial trait described by Eysenck and Eysenck (1968), which presents important correlations with psychopathy as a trait, defining the perpetrators as more violent, impulsive, and less empathetic than their victims (Galán et al., 2023).

Contrasting the IPF victims with the non-lethal ones, there were no significant differences in any of the personality dimensions convergent with Ulloa et al. (2016) findings. Considering, as stated before that the personality differences are bigger between the perpetrators than between the victims of the different groups, the personality pattern of the perpetrator should be understood as a risk factor for IPF and considered by practitioners at the moment they receive an IPVAW report to extreme their precautions.

## Limitations and Conclusions

The first limitation affects study 2. The objectivity of the method used for personality assessment, indirect profiling, can be affected by many risks or biases such as cultural or political differences, prejudices, or other personal variables (Back & Nestler, 2016; Meloy, 2004). To overcome this limitation, study 1 was performed, showing that the interviewers were able to perform accurate judgements. However, since the main objective of the present study was to assess the perpetrator’s and victims’ personalities, no information was recorded on how to better retrieve these observational cues, which we consider a very interesting area of investigation in personality assessment. In addition, with regard to study 1, the nature of the sample prevented the inclusion of a control group for assessing whether accurate judgements were influenced or biased by interviewers’ backgrounds, training effects, or other variables, such as the timing of the completion of the EPQR-A questionnaire, which consistently occurred after the interviews (Funder, 2012; Karelaia & Hogarth, 2008).

A second limitation is related to the correlational methods. Since the groups, although similar, were not matched attending to different sociodemographic variables, thus the personality differences might have been affected by variables like the number of stressors at the time of the events, lack of support from the perpetrator, or other variables that differentiate these groups (for more information about these differences, see Pineda, Galán, et al., 2023; Pineda, Rico-Bordera, et al., 2023).

A third limitation pertains to the group of IPF victims and the constraint of assessing them solely through the psychological autopsy method. While this approach is reliable, a more comprehensive evaluation is always preferable, ideally employing various direct and indirect methodologies. This becomes particularly important, especially when the bulk of the information about the deceased individuals is derived from external third-party perspectives (Aquila et al., 2018; Isometsä, 2001).

Finally, attending to the representativeness of the sample, since the participants were not receiving any compensation for taking part in the study, some of the randomly contacted participants denied participating. In those cases, a new couple was randomly selected attending to the Spanish foreigners proportions mentioned.

In conclusion, we have provided information for police officers and other practitioners the moment they receive an IPVAW report to extreme their precautions based on the personality of the perpetrators. In this sense, IPF perpetrators tend to be less emotionally stable (i.e. high neuroticism), with a lower interest in having social interactions with other people or more interested in their own thoughts and feelings (i.e. low extraversion) while also presenting a tendency towards bizarrely, as well as being more aggressive or less empathic (i.e. high psychoticism). These findings can also help practitioners in developing more specific reinsertion programs attending to the specific population differences found in these groups mainly focusing on controlling the behaviours related to the psychoticism trait (Costa & McCrae, 1994). Furthermore, we have found that personality typology in all victims and the non-lethal IPV perpetrators is more diffuse than in the IPF group. However, the victims tend to appear as more extroverted, more emotionally stable, and with lower scores in psychoticism compared with the IPF perpetrators.

## Data Availability

The data that support the findings of this study are openly available in osf.io at 10.17605/OSF.IO/VM9XC.
